# Proteomic Profiling of Primary Human Acute Myeloid Leukemia Cells Does Not Reflect Their Constitutive Release of Soluble Mediators

**DOI:** 10.3390/proteomes7010001

**Published:** 2018-12-20

**Authors:** Elise Aasebø, Maria Hernandez-Valladares, Frode Selheim, Frode S. Berven, Annette K. Brenner, Øystein Bruserud

**Affiliations:** 1 Department of Clinical Science, Section for Hematology, University of Bergen, 5021 Bergen, Norway; Elise.Aasebo@uib.no (E.A.); Maria.Hernandez-Valladares@uib.no (M.H.-V.); Annette.Brenner@uib.no (A.K.B.); 2 Department of Biomedicine, The Proteomics Unit at the University of Bergen (PROBE), University of Bergen, 5009 Bergen, Norway; Frode.Selheim@uib.no (F.S.); Frode.Berven@uib.no (F.S.B.); 3 Department of Medicine, Haukeland University Hospital, 5021 Bergen, Norway

**Keywords:** acute myeloid leukemia, cytokine, protease, constitutive secretion, proteomics

## Abstract

Acute myeloid leukemia (AML) is a heterogeneous disease, and communication between leukemic cells and their neighboring leukemia-supporting normal cells is involved in leukemogenesis. The bone marrow cytokine network is therefore important, and the mediator release profile seems more important than single mediators. It is not known whether the characterization of primary AML cell proteomes reflects the heterogeneity of the broad and dynamic constitutive mediator release profile by these cells. To address this, we compared the intracellular levels of 41 proteins in 19 AML patients with the constitutive extracellular release during in vitro culture, including chemokines, growth factors, proteases, and protease regulators. The constitutive release of most mediators showed a wide variation (up to 2000-fold differences) between patients. Detectable intracellular levels were seen for 10 of 41 mediators, but for most of these 10 mediators we could not detect significant correlations between the constitutive release during in vitro culture and their intracellular levels. Intracellular protein levels in primary human AML cells do not reflect the dynamics, capacity, and variation between patients in constitutive mediator release profiles. Measurements of these profiles thus add complementary information to proteomic detection/quantification regarding the heterogeneity of the AML cell contributions to the bone marrow cytokine network.

## 1. Introduction

Acute myeloid leukemia (AML) is an aggressive malignancy characterized by the infiltration of immature leukemia blasts in the bone marrow [1]. Several nonleukemic bone marrow cells support the development of the disease, including the various cells of the stem cell niches that support the maintenance of the most immature leukemic stem cells [2,3]. The bidirectional crosstalk between leukemic and non-leukemic cells is mediated both by direct cell–cell contact and through the release of soluble mediators into their common bone marrow microenvironment [4,5,6]. The constitutive release (here defined as mediators secreted without specific stimuli) of a wide range of soluble mediators by the primary human AML cells is thought to be an important part of this crosstalk. This release is also important for autocrine loops and, thereby, the spontaneous proliferation of AML cells, and the ability of autocrine proliferation is also associated with the prognosis [7].

AML is a very heterogeneous disease [1], and proteomic investigations can be used to further characterize this heterogeneity [8,9,10,11]. A wide variation in the constitutive release of several soluble mediators is a part of this patient heterogeneity [12,13]. Previous studies of this release have been based on the detection of extracellular mediator levels after in vitro culture of enriched AML cells under highly standardized conditions, i.e., evaluation of mediator release in a dynamic model, and the differences between patients in this assay are even associated with prognosis. However, this possible prognostic impact needs to be further investigated, but biological characteristics detected during in vitro culture are not suitable for implementation in clinical studies or routine clinical practice. For these reasons, we have investigated whether an analysis of intracellular mediator levels, i.e., the snapshot of intracellular proteins at the time of sample collection, reflects the dynamic process of constitutive mediator release from these cells. We then compared the proteomic quantitation of cellular soluble mediators in enriched primary AML cells with the constitutive extracellular mediator release during standardized in vitro culture of the leukemic cells.

## 2. Materials and Methods

### 2.1. Collection and Preparation of Primary AML Patient Cells

The study was approved by the local Ethics Committee (Regional Ethics Committee III, University of Bergen; REK 2013/634), and samples were collected after written informed consent. AML blasts were derived from 19 unselected patients admitted to our hospital for treatment of acute myeloid leukemia (7 females and 12 males; median age 52 years with range 18–68 years). The present study included all patients from a defined geographical area and during a defined time period who were below 70 years of age, had circulating AML blasts in the peripheral blood, and were regarded to be fit for intensive (i.e., potentially curative) antileukemic treatment. The patient characteristics are summarized in Table 1. A major part of the patients had de novo AML with normal karyotype. Primary AML cells were isolated from peripheral blood by density gradient separation (Lymphoprep; Axis-Shield, Oslo, Norway; specific density 1.077 g/mL). All patients showed high levels (>20 × 10^9^/L) and/or percentage (>80%) of circulating AML blasts, and a highly enriched blast population could thereby be prepared by density gradient separation alone [14,15,16]. The prepared leukemic cell populations contained at least 90% blasts and the cells were stored in liquid nitrogen until used. 

### 2.2. Protein Analyses and In Vitro Culture

Primary AML cells from all 19 patients were thawed and immediately processed for proteomic analysis/quantification according to the sample preparation protocol previously described in detail [8,11]. Briefly, the cells were lysed in 4% SDS/0.1M Tris-HCl pH 7.6, and 20 µg protein samples were prepared according to the filter-aided sample preparation protocol [17]. For proteomic analysis, approximately 1 µg per sample, dissolved in 2% acetonitrile (ACN) and 1% formic acid (FA), was injected into an Ultimate 3000 Rapid Separation LC system (Thermo Scientific, Sunnyvale, California, CA, USA) coupled to a QExactive HF mass spectrometer (MS) (Thermo Scientific, Bremen, Germany) equipped with an EASY-spray ion source (Thermo Scientific). The peptides were pre-concentrated on a precolumn (Acclaim PepMap100, 2 cm × 75 µm I.D. nanoViper column, packed with 3 µm C18 beads with pore size 100Å), and separated on a 50 cm-long analytical column (Acclaim PepMap RSLC, 50 cm × 75 µm I.D. EASY-spray column, packed with 2 µm C18 beads with pore size 100Å) at a flow rate of 200 nL/min. The peptides were eluted by a binary gradient of solvent A (0.1% FA) and solvent B (0.1% FA/ACN) with the following 195 min gradient composition: 5% B during trapping from 0 to 5 min followed by increase to 8% B from 5 to 5.5 min, then to 24% B from 5.5 to 115 min, to 35% B from 115 to 140 min, and to 90% B from 140 to 155 min. Hold at 90% B for 15 min and ramped to 5% B from 170 to 195 min. 

The QExactive HF was operated in data-dependent acquisition (DDA) mode, thus switching between survey full scan MS and MS/MS acquisition. The full scans (from *m*/*z* 375–1500) were acquired in profile mode with a resolution of R = 120,000 at 200 *m*/*z*, an automatic gain control (AGC) target value of 3 × 10^6^ and maximum injection time (IT) of 100 ms. The top 12 precursors (above the intensity threshold of 5 × 10^4^ counts) were sequentially isolated for MS/MS. The scans were acquired in centroid mode with a resolution of R = 30,000, an AGC target value of 1 × 10^5^ and a maximum IT of 110 ms. Normalized collision energy was set to 28%, the isolation window was 1.6 *m*/*z* (offset 0.3 *m*/*z*) and the dynamic exclusion lasted for 25 s. Furthermore, for spray and ion-source parameters, the ion spray voltage was at 1800 V, no sheath and auxiliary gas flow, and the capillary temperature was at 260 °C.

The raw MS files were searched in MaxQuant (v.1.5.6.0) [18] using default parameters, with the following exceptions: Gln conversion to pyro-Glu was included as a variable modification, trypsin was used for proteolytic digestion, peptide length was set to 6 amino acids, the match-between-runs option was enabled, and the minimum ratio count was set to 1 for label-free quantitation (LFQ) (i.e., the same peptide needs to be present in at least two samples for quantification). The cellular protein levels were relatively quantified using the MaxLFQ algorithm [19], and these intracellular levels are presented as the relative LFQ intensity defined as the normalized relative protein abundance compared across the MS runs. 

Our in vitro model for investigation of constitutive mediator release has been previously described in detail [12,13,20]. Briefly, supernatants were collected from all patients’ AML cells after being cultured alone for 48 h in Stem Span SFEMTM medium (Stem Cell Technologies; Vancouver, BC, Canada) in 24-well culture plates (1 × 10^6^ cells per mL; Nunclon, Roskilde, Denmark). The levels of 41 mediators were analyzed either by Luminex assays or ELISA analyses, as described previously [12,13,21]. The raw data of constitutive mediator release for the 19 patients of interest were extracted from our previous studies comprising 79 patients, in which 7 of the 19 patients were identified with low, 4 with intermediate, and 8 with high mediator release profiles [13]. 

Spearman’s test was used to assess the correlation between the intracellular and extracellular levels of the mediators (GraphPad Prism version 7.00, California, CA, USA).

## 3. Results and Discussion

AML is a bone marrow disease, and the constitutive release of soluble mediators by leukemic cells is important for the communication between these cells and their neighboring AML-supporting cells in the common bone marrow microenvironment [4,5,22,23,24]. The constitutive release may even have a prognostic impact [13], and one can therefore argue that this parameter should be further evaluated in future clinical studies as a possible prognostic factor. However, in vitro culture is not suitable as a biomarker in clinical studies or routine clinical practice, and an important question is therefore whether a snapshot examination of intracellular mediator levels reflects and can replace the more complex analysis of the dynamic constitutive release, the patient heterogeneity, and the complexity of the release profile. We investigated the release of a wide range of mediators in our previously established and standardized in vitro culture model, where the prognostic impact was observed, and we compared the results from this model with the analyses of intracellular mediator levels. Thus, our main intention in the present study was to investigate whether snapshot analysis of intracellular mediator levels reflects the dynamic process of extracellular release. A proteomic approach offers the possibility to analyze a large number of cellular proteins to analyze the intracellular mediator profile by itself and as an interacting part of a complex biological context. We therefore explored whether such an intracellular snapshot reflects the dynamics (i.e., variation between patients, complexity of the release profile) of the constitutive release that is seen in the in vitro culture model. 

We investigated the constitutive release of 41 soluble mediators by in vitro cultured primary human AML cells derived from 19 patients; for 38 of them, detectable release was seen for a majority of patients, while ADAM12 had undetectable levels in all patients. The overall results are summarized in Supplemental Table 1. It can be seen that the large majority of mediators showed a wide variation among patients, and the fold variation between patients with detectable release was more than 2000-fold for several mediators. Investigation of the technical variation (data not shown) between duplicate determinations showed <10% and usually <5% variation, and can thus not explain the large variation between the patients. Furthermore, all samples were investigated in the same ELISA or Luminex assay to avoid inter-assay variations. Lastly, the reproducibility of measurements of 19 mediators in independent experiments (i.e., thawing of a second ampulla, separate cell culture preparations, and separate mediator analyses) was assessed for 13 random patients from the biobank. We observed strong correlations between mediator levels detected in our present article, and levels detected in these completely independent experiments for 17 of the 19 tested mediators (*r*-values >0.49 and *p*-values < 0.04). For 13 of these 19 mediators the *r*-values were >0.60. 

We identified 5790 protein groups in our proteomic data after removing reverse hits and proteins only identified by site. The number of quantified proteins per patient varied from 3823 to 5102. Of these, 5777 proteins had a quantitative value, and only 10 of the 41 mediators of interest were identified/quantified (Table 2), indicating low intracellular concentration for the majority of the 41 mediators compared to many other cellular proteins. The number of peptides quantified per protein varied among the patient samples: BSG, CASP1, ELANE were quantified with two or more peptides in every patient sample, while CCL5 was quantified with one or two peptides in the seven patient samples with measurable abundance (Supplemental Table S2). The peptides quantified for MMP-2 were also present in the sequence of MMP-7, hence, MMP-2 was not considered as quantified. The ten mediators that could be quantified included nine proteases/protease regulators and only one chemokine (CCL5). Thus, of 41 the selected mediators in Supplemental Table S1, only one of eleven chemokines was quantified, while none of the six interleukins or the eight growth factors was quantified among the many thousands of proteins from the proteomics approach. Constitutive CCL5 release by the in vitro cultured AML cells was detected for all 19 patients in the Luminex assay, whereas it was only detected for 7 patients with the proteomic approach. BSG was the only protein detected in all 19 patients with both approaches. The fact that only one chemokine and nine proteases/protease regulators were found intracellularly (using proteomic quantification), while 38 of our selected mediators were detected extracellularly (in the supernatants after 48 h in vitro cell culturing), may suggest that many chemokines, growth factors, and interleukins are of low abundance intracellularly, and thus challenges protein detection with our proteomic procedure.

**Figure 1:**
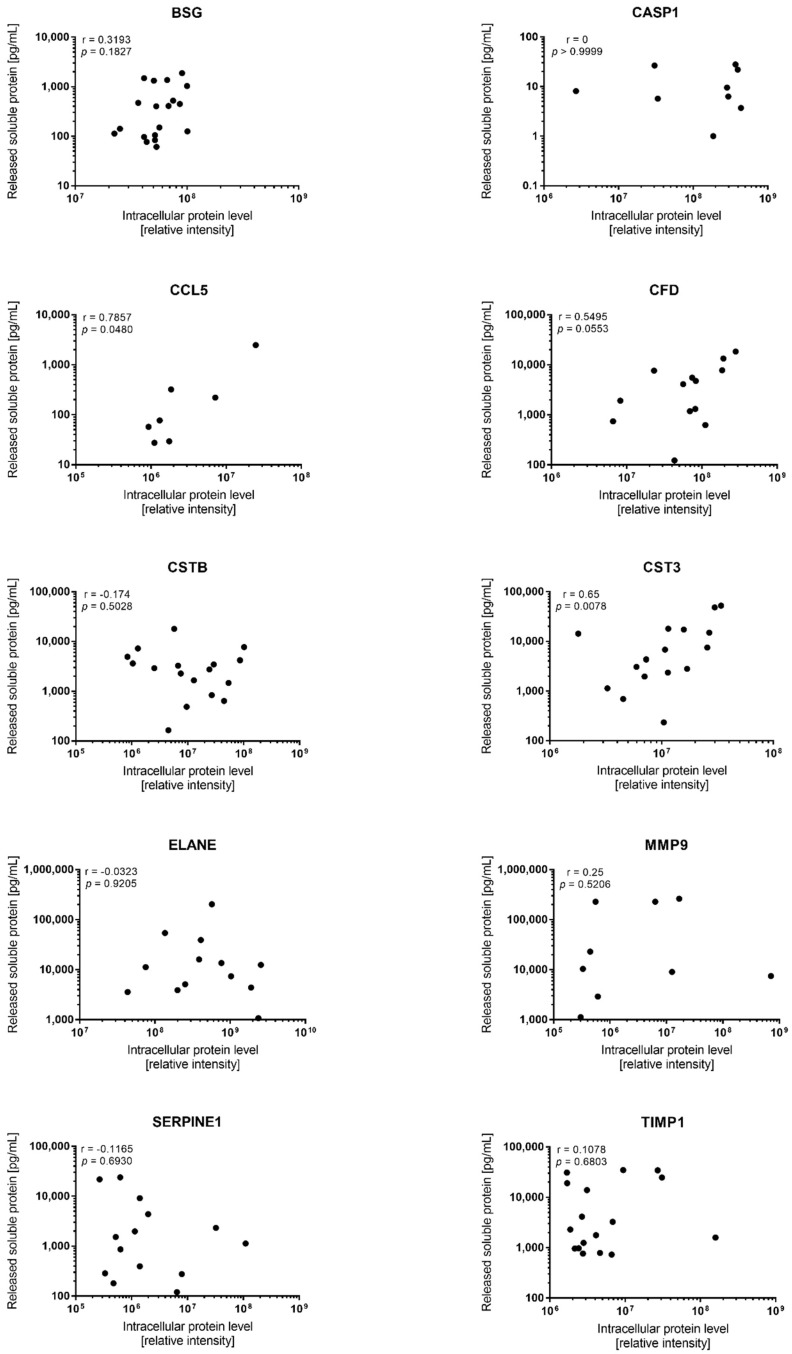
Comparison of constitutive mediator release during in vitro culture and the corresponding proteomic abundance of cellular protein levels (relative intensity). Cellular levels in the proteomic analysis were quantified for 10 of the 41 mediators; this included the chemokine CCL5 (quantified intracellular levels for 7 patients/detectable extracellular release during culture for 19 patients) together with the proteases or protease regulators CFD (18/13 patients), TIMP1 (17/19 patients), MMP9 (13/12 patients), serpin E1 (14/19 patients), BSG (19/19 patients), cystatin B (18/18 patients), cystatin C (16/19 patients), neutrophil elastase /ELANE (19/13 patients), and caspase 1 (19/9 patients). The figure presents the results for all mediators showing quantifiable levels in the proteomic analysis, and the results are presented as the intracellular protein level (relative label-free quantitation (LFQ) intensity, x-axis) versus the corresponding supernatant concentrations (i.e., released soluble protein, pg/mL, y-axis). The corresponding Spearman *r*- and *p*-values are indicated in the figure for each of the 10 mediators.

Proteomic analysis of the AML cells allows identification and quantification of the proteins and their relative abundance in a particular compartment at a specific time. This analysis therefore represents a static snapshot of ongoing biological processes. By exploring the correlation of intracellular protein abundances with the concentration released into the media after 48 h in vitro culturing, we intended to investigate the dynamics of mediator release in the 19 AML patients (Figure 1). 

The question of interest is what the different correlation analyses of these soluble mediators reflect. There are three expected outcomes of the analyses: (1) correlation of intracellular and extracellular levels, indicating a steady relationship between protein synthesis and mediator release; (2) high intracellular levels and low constitutive release of soluble mediators, indicating mainly intracellular protein functions; or (3) low intracellular levels and high constitutive release of soluble mediators, indicating rapid mediator release for extracellular functions. A correlation between the in vitro culture and ex vivo proteomic levels was only seen for CCL5, CFD, and cystatin C/CST3; a highly significant correlation was seen for cystatin C/CST3 (*p* = 0.0078, *r* = 0.65) whereas only borderline significance was reached for CCL5 (*p* = 0.048, *r* = 0.79) and CFD (*p* = 0.055, *r* = 0.55). For the seven other mediators, the *p*-values did not obtain statistical significance, and *r*-values were generally <0.25 (Figure 1). Interestingly, for several of these mediators (e.g., TIMP1 and cystatin B), we observed correlation plots with a combination of the expected outcomes, potentially reflecting the large heterogeneity among the patients. For example, the intracellular and released levels of TIMP1 seem to correlate for some patients, however, while it was not quantified intracellularly for one patient with high released levels, it had the highest intracellular levels in another patient of which the released level was below the median (Supplemental Table S1). By contrast, cystatin B seems to be more heterogeneous at the intracellular level compared to the released level, although the fold variation (i.e., between the highest and lowest level) is relatively similar for both assays. A subset of the patients demonstrated large variations in their constitutive release of serpin E1, while the intracellular levels in these particular patients were relatively similar. 

We hypothesize that the lack of correlation between intracellular and released extracellular levels for most mediators indicates that the intracellular levels, at least for certain mediators, reflect additional intracellular functions rather than the capacity of extracellular release. This may also be the explanation for the similar lack of correlation between intracellular and extracellular levels of osteopontin [25]. At the same time, higher extracellular release may represent a different phenotype with a more pronounced cell–cell communication. With exception of CCL5, the detected mediators are all proteases and protease regulators. VEGF shows both intra- and extracellular autocrine signaling [26], and the same may also be true for other soluble mediators. Several matrix metalloproteases, various TIMPs, CFD, and cystatins, seem to be important for intracellular functions with regard to signaling, regulation of apoptosis, and regulation of mitochondrial or nuclear functions [27,28,29,30,31,32,33,34,35,36]. 

To decrease the risk of inducing biological alterations in the leukemic cells by more extensive cell separation procedures [15,16], we only investigated patients with a high peripheral blood blast count/percentage. We therefore emphasize that our present observations may be representative only for this particular patient subset with relatively high peripheral blood blast counts.

It has been suggested by others that cryopreserved AML cells should be cultured in vitro for a short period before proteomic analysis to reduce the effects of the cryopreservation-induced stress on the leukemic cells [37]. However, additional in vitro culture will induce the process of spontaneous or in vitro induced apoptosis in primary AML cells [38], and this ongoing biological process may affect the proteome. To avoid the influence of this additional factor and to analyze a sample condition more likely to be used in clinical practice, we investigated the leukemic cells immediately after thawing in our proteomic studies, but it should be noted that the in vitro cultured cells may differ from these cells. 

We have previously shown that cryopreservation and thawing of primary human AML cells will alter the proteome and phosphoproteome [8,39], including proteins involved in the mitotic processes and signaling downstream to lectin receptors and CLEC7A signaling, that was reduced in cryopreserved cells [8]. However, although other patient samples were used in our previous studies, none of the proteins explored here (and quantified in our previous proteomic studies) were significantly altered according to the applied statistics [8]. Thus, in our opinion, it is less likely that the lack of correlation between proteomic and in vitro culture levels are caused by cryopreservation-induced alterations.

Our previous studies suggest that the capacity of constitutive mediator release is associated with a favorable prognosis [13], and it may therefore be of interest to evaluate the constitutive release in future clinical studies to clarify whether it is an independent prognostic parameter or only a part of a more complex phenotype that is associated with already identified independent prognostic markers. In vitro culturing is not suitable for routine clinical practice, and analysis of mediator expression at the mRNA level is possibly not reliable because there is not always a correlation between mRNA expression and protein level/secretion [12,13]. Our preliminary results suggest that mRNA and protein expression of genes that are important for transcriptional regulation and intracellular organellar functions/trafficking (especially the V-ATPase complex) may reflect constitutive cytokine release, and may thereby be used as a biomarker for this functional characteristic [13].

## 4. Conclusions

Communication between malignant and nonmalignant neighboring cells through the cytokine network is important in AML. AML cells show constitutive release of a wide range of soluble mediators, and the overall release profile or combined effects of several mediators may then be more important than the effects of single mediators. Our study shows that for most mediators, the ability of constitutive extracellular release by primary human AML is best evaluated by in vitro culture, and not by analyzing intracellular levels as a static representation of the potential secretome. However, several soluble mediators demonstrate a wide variation between patients both intracellularly and when constitutively released, thus reflecting the large heterogeneity in AML. A combination of different methodological strategies may be essential to capture this heterogeneity and for a deeper understanding of leukemogenesis and AML cells’ communication with the microenvironment.

## Figures and Tables

**Table 1:** Biological and clinical characteristics of the 19 AML patients included in the study.

Patient Characteristics		Cell Morphology		Cell Genetics	
Age		FAB classification		Cytogenetics	
Median (years)	52	M0	1	Favorable	3
Range (years)	18–68	M1	5	Intermediate	1
		M2	3	Normal	13
Gender		M4	4	Adverse	2
Females	7	M5	6		
Males	12			*Flt3 mutations*	
				ITD	9 ^1^
Disease		CD34 receptor		Wild-type	10
De novo AML	14	Negative (≤20%)	13		
Secondary AML	2	Positive (>20%)	6	NPM1 mutations	
AML relapse	3			Insertion alone	4
				Wild-type	12
				Insertion+Flt3-ITD	3

^1^ One of these patients has an additional point mutation at D835.

**Table 2:** Soluble mediator levels detected during constitutive in vitro culture and by the proteomic analysis. The table shows a comparison for the 10 soluble mediators that showed quantifiable levels in the proteomic analysis. For the constitutive mediator release during in vitro culture studies, we present the number of patients with detectable levels, median level (pg/mL), variation range, and fold variation (i.e., the fold change between the highest and lowest quantitative value in each assay) for each of the mediators, measured with Luminex/ELISA. The proteomic results are presented as the number of patients with quantified levels and the variation among these patients. The results from the statistical analyses (Spearman’s test, ns means not statistically significant) are presented in the right column.

Mediator	Detectable Constitutive Release During in Vitro Culture				Quantifiable Levels in Proteomic Analysis		Correlation (*p*-Value)
	Number	Median	Range	Fold Variation	Number	Fold Variation	
CCL5	19	56.3	17.4–2481	143	7	27	0.0480
MMP9	12	23,957	1123–261,935	233	13	2361	ns
TIMP1 (tissue inhibitor of MMPs)	19	2298	580–34,789	60	17	95	ns
Caspase 1/CASP1	9	8.1	1.0–28.0	28	19	163	ns
BSG (basigin)	19	401	61.2–1876	31	19	4.5	ns
CFD (complement factor D)	13	4106	123–18,426	150	18	139	0.0553
Cystatin B/CSTB	18	2846	165–17,949	109	18	121	ns
Cystatin C/CST3	19	3075	233–52,208	224	16	19	0.0078
Neutrophil elastase/ELANE	13	11,224	1072–203,665	190	19	835	ns
Serpin E1	19	1131	36.2–23,925	661	14	412	ns

## Supplementary Materials

The following are available online http://www.mdpi.com/2227-7382/7/1/1/s1, Supplemental Table S1: Levels of constitutive cytokine release during in vitro culture by enriched primary human AML cells. For each mediator, we present the number of patients (*n* = 19 patient samples were included) with detectable levels, together with the median level (pg/mL) and the range for those patients showing detectable levels when measured with Luminex/ELISA assays. The mediators showing detectable levels in the proteomic examination are marked with an asterisk to the left in the figure. Supplemental Table S2: Proteomic LFQ results for the relevant proteins, and concentration of the constitutively released mediators.
