# Supplementary material for: Proteomic Profiling of Primary Human Acute Myeloid Leukemia Cells Does Not Reflect Their Constitutive Release of Soluble Mediators
Source: Proteomes. 2018 Dec 20;7(1):1. doi: 10.3390/proteomes7010001 (PMC6473519; doi:10.3390/proteomes7010001)
Supplement: Supplementary file 1 [file proteomes-07-00001-s001.zip › Supplemental Table 1-SA.pdf]

**Supplemental Table 1. Levels of constitutive cytokine release during *in vitro* culture by enriched primary human AML cells.** For each mediator we present the number of patients (n=19 patient samples were included) with detectable levels together with the median level (pg/mL) and the range for those patients showing detectable levels when measured with Luminex/ELISA assays. The mediators showing detectable levels in the proteomic examination are marked with an asterisk to the left in the figure.

| | Mediator | Number | Median | Range | Fold variation |
|---|---|---|---|---|---|
| Chemokines (n=11) | | | | | |
| | CCL2 | 19 | 262 | 1.4-8202 | 5859 |
| | CCL3 | 19 | 451 | 131->30,000 | >229 |
| | CCL4 | 19 | 254 | 45.9-11,744 | 256 |
| * | CCL5 | 19 | 56.3 | 17.4-2481 | 143 |
| | CCL28 | 5 | 123 | 53.8-432 | 8 |
| | CXCL1 | 19 | 110 | 51.5-3520 | 68 |
| | CXCL2 | 17 | 21.1 | 2.3-9057 | 3938 |
| | CXCL5 | 18 | 313 | 6.6-13,980 | 2118 |
| | CXCL8 | 19 | 1676 | 1.8->18,500 | >10,278 |
| | CXCL10 | 19 | 11.7 | 1.6-15,504 | 9690 |
| | CXCL11 | 19 | 91.4 | 62.5-246 | 3.9 |
| Interleukins - immunoregulators (n=6) | | | | | |
| | IL1β | 19 | 4.4 | 1.2-763 | 636 |
| | IL1RA | 19 | >10,750 | 7.5->10,750 | >1433 |
| | IL6 | 10 | 142 | 1.5-4335 | 2890 |
| | IL10 | 19 | 1.6 | 1.3-7.4 | 5.7 |
| | IL33 | 19 | 8.9 | 7.7-22.0 | 2.9 |
| | TNFα | 19 | 11.8 | 0.8-692 | 865 |
| Growth factors (n=8) | | | | | |
| | Angiopoietin 1 | 14 | 130 | 7.2-454 | 63 |
| | TEK/Tie-2 (angiopoietin receptor) | 19 | 190 | 121-248 | 2 |
| | FGF2 (fibroblast growth factor) | 19 | 19.6 | 14.2-23.9 | 1.7 |
| | G-CSF (granulocyte colony-stimulating factor) | 6 | 15.2 | 0.22-60.5 | 275 |
| | GM-CSF (granulocyte-macrophage CSF) | 19 | 12.4 | 10.3-555 | 54 |
| | HGF (hepatocyte growth factor) | 19 | 36.9 | 5.9-506 | 86 |
| | Hb-EGF (Proheparin-binding EGF-like growth factor) | 19 | 17.7 | 4.7-70.8 | 15 |
| | VEGF (vascular endothelial growth factor) | 19 | 12.3 | 6.7-51.6 | 7.7 |
| Proteases and protease regulators (n=14) | | | | | |
| | MMP1 (matrix metalloprotease 1) | 19 | 40.3 | 28.7-4552 | 159 |
| | MMP2 | 19 | 1981 | 367-9485 | 26 |
| * | MMP9 | 12 | 23,957 | 1123-261,935 | 233 |
| * | TIMP1 (tissue inhibitor of MMP 1) | 19 | 2298 | 580-34,789 | 60 |
| * | Caspase 1 | 9 | 8.1 | 1.0-28.0 | 28 |
| * | BSG (basigin) | 19 | 401 | 61.2-1876 | 31 |

| | | | | | |
|---|---|---|---|---|---|
| * | CFD (complement factor D) | 13 | 4106 | 123-18,426 | 150 |
| * | Cystatin B/CSTB | 18 | 2846 | 165-17,949 | 109 |
| * | Cystatin C/CST3 | 19 | 3075 | 233-52,208 | 224 |
| * | Neutrophil elastase/ELANE | 13 | 11,224 | 1072-203,665 | 190 |
| * | Serpin E1 | 19 | 1131 | 36.2-23,925 | 661 |
| | Serpin C1 | 19 | 61,579 | 52,373-64,196 | 1.2 |
| | SLPI (antileukoproteinase) | 7 | 19 | 6-163 | 27 |
| | uPA (urokinase-type plasminogen activator) | 19 | 373 | 53.4-2438 | 46 |

ADAM12 (proteases A disintegrin and metalloprotease domain 12) and ADAMTS 13 (ADAM with thrombospondin type 1 motif member 13) showed detectable levels for none and for 2 patients, respectively.
