# Supplementary material for: Proteomic Profiling of Primary Human Acute Myeloid Leukemia Cells Does Not Reflect Their Constitutive Release of Soluble Mediators
Source: Proteomes. 2018 Dec 20;7(1):1. doi: 10.3390/proteomes7010001 (PMC6473519; doi:10.3390/proteomes7010001)
Supplement: Supplementary file 1 [file proteomes-07-00001-s001.zip › Supplemental Table 2.pdf]

	CFD (complement factor D) (Acc. no P00746)			TIMP-1 (Acc. No P01033)			Cystatin C/CST3 (Acc. No P01034)			Cystatin B/CSTB (Acc. No P04080)			Serpin E1 (Acc. No P05121)		
	Proteomics (intracellular level)		Constitutively released	Proteomics (intracellular level)		Constitutively released	Proteomics (intracellular level)		Constitutively released	Proteomics (intracellular level)		Constitutively released	Proteomics (intracellular level)		Constitutively released
Patient code	#Peptides	LFQ intensity	Concentration (pg/mL)	#Peptides	LFQ intensity	Concentration (pg/mL)	#Peptides	LFQ intensity	Concentration (pg/mL)	#Peptides	LFQ intensity	Concentration (pg/mL)	#Peptides	LFQ intensity	Concentration (pg/mL)
P1	8	111320000	627	1	2749900	767	0	n.d.	693.5	5	44701000	640	0	n.d.	136.5
P11	6	23164000	7651	1	1680900	30946	1	11481000	17964.1	4	24444000	2769	1	627590	23924.9
P12	4	8225000	1923	2	2666100	4132	1	7266000	4321.6	4	9591100	487	1	1147200	1983.2
P14	7	281450000	18426	0	n.d.	25149	1	34039000	52208.4	2	5742900	17949	0	n.d.	2269.2
P17	3	6586000	744	7	159080000	1587	0	n.d.	2240.6	2	2453100	n.d.	7	110160000	1130.7
P18	9	69352000	1182	1	2796900	1248	3	16922000	2813.9	1	1286400	7238	1	335550	286.3
P19	8	81825000	1318	1	1863400	2298	1	7041600	1963.2	4	29251000	3479	1	630960	864.9
P2	3	7627700	n.d.	1	4100500	972	1	11382000	2366.8	3	1046200	165	0	521950	1527.7
P29	5	2017600	n.d.	2	2415400	1770	1	10723000	6768	1	4515700	3648	1	n.d.	36.2
P30	1	3382500	n.d.	3	4635900	788	0	n.d.	693.5	1	839670	4914	1	474040	180.7
P32	0	n.d.	n.d.	1	2141100	964	1	4534600	693.5	4	6714900	3275	5	7933600	276.4
P36	7	56240000	4106	4	27047000	34315	2	25588000	7472.2	5	101570000	7674	2	1405000	9151.5
P38	9	74302000	5555	2	3106500	13893	1	1796300	14288.7	4	53823000	1477	0	n.d.	3387.8
P39	8	83161000	4765	2	1693700	19059	2	15778000	17196	5	12871000	1661	1	267640	21867.9
P44	9	192940000	13337	3	9416200	34789	3	29835000	48278.9	5	85942000	4200	3	1982200	4378.3
P46	9	185940000	7797	6	30896000	24776	2	26669000	14959.7	5	26927000	837	6	32372000	2320.8
P48	8	43303000	123	3	6838300	3260	1	3277100	1137	2	2527500	2923	3	1402800	394.5
P6	4	7799100	n.d.	0	n.d.	580	2	10458000	233.3	0	n.d.	831	0	n.d.	70.5
P9	3	5530800	n.d.	1	6618600	731	1	5963000	3075.4	4	7537800	2285	4	6481200	120.1
# valid values		18	13		17	19		16	19		18	18		14	19
Max value		281450000	18426		159080000	34789		34039000	52208.4		101570000	17949		110160000	23925
Min value		2017600	123		1680900	580		1796300	233.3		839670	165		267640	36.2
Fold change (Max-Min)		139	150		95	60		18.9	224		121	109		412	661
Median		49771500	4106		3106500	2298		11052500	3075		8564450	2846		1275000	1131

	Neutrophil elastase/ELANE (Acc. No P08246)			CCL5 (Acc. No P13501)			MMP-9 (Acc. No P14780)			Caspase 1 (Acc. No P29466)			BSG/CD147 (Acc. No P35613)		
	Proteomics (intracellular level)		Constitutively released	Proteomics (intracellular level)		Constitutively released	Proteomics (intracellular level)		Constitutively released	Proteomics (intracellular level)		Constitutively released	Proteomics (intracellular level)		Constitutively released
Patient code	#Peptides	LFQ intensity	Concentration (pg/mL)	#Peptides	LFQ intensity	Concentration (pg/mL)	#Peptides	LFQ intensity	Concentration (pg/mL)	#Peptides	LFQ intensity	Concentration (pg/mL)	#Peptides	LFQ intensity	Concentration (pg/mL)
P1	7	253330000	5102	0	n.d.	17.4	4	1454500	n.d.	23	277290000	nd	6	41281000	97
P11	11	1904100000	4415	0	n.d.	323.7	17	16808000	261935	22	295610000	6.3	7	41322000	1486.5
P12	8	408080000	39348	0	n.d.	27.1	0	n.d.	24963	18	103560000	nd	5	25181000	141.7
P14	10	135910000	54207	1	1749900	29.5	1	444510	22950	17	185600000	1	5	66563000	1368.2
P17	7	27912000	n.d.	2	24813000	2481.3	0	n.d.	n.d.	17	77101000	nd	7	56551000	149.8
P18	11	365720000	n.d.	0	n.d.	43.1	1	301130	1123	11	30339000	26.5	6	68396000	409.6
P19	11	2567300000	12483	1	929370	57.5	26	711020000	7493	23	284800000	9.5	6	53072000	401.2
P2	6	560330000	3583	0	n.d.	56.1	1	606050	n.d.	20	33600000	5.7	5	75183000	522.3
P29	10	43351000	n.d.	0	n.d.	20.3	3	609010	2909	12	131000000	nd	5	51737000	105.3
P30	7	44229000	n.d.	1	1853700	320.9	12	10232000	n.d.	2	2690800	8.1	2	51736000	83.2
P32	10	199980000	3907	1	1110400	27.4	11	5667700	n.d.	11	27822000	nd	5	43596000	77.1
P36	9	2381400000	1072	0	n.d.	348.5	12	6339700	228146	20	369870000	28	8	90374000	1875.9
P38	10	572250000	203665	0	n.d.	353.8	1	330690	10410	23	333290000	nd	5	36622000	472.1
P39	9	1670600000	n.d.	0	n.d.	119.2	0	n.d.	58237	21	366730000	nd	6	50568000	1325.2
P44	9	766680000	13613	1	1306100	76.8	0	n.d.	200178	24	437300000	3.7	7	100050000	1029.9
P46	9	387130000	16074	1	7171500	220.7	3	552720	229058	25	395880000	21.9	7	86326000	447.1
P48	11	1027500000	7380	0	n.d.	56.3	18	12529000	9067	13	71655000	nd	4	101210000	125.5
P6	6	75296000	11224	0	n.d.	54.1	0	n.d.	n.d.	6	10606000	nd	4	22437000	113.3
P9	2	3073300	n.d.	0	n.d.	43.6	0	n.d.	n.d.	17	57092000	nd	5	53359000	61.2
# valid values		19	13		7	19		13	12		19	9		19	19
Max value		2567300000	203665		24813000	2481		711020000	261935		437300000	28.0		101210000	1875.9
Min value		3073300	1072		929370	17.4		301130	1123		2690800	1.00		22437000	61.2
Fold change (Max-Min)		835	190		27	143		2361	233		163	28.0		4.5	30.7
Median		387130000	11224		1749900	56.3		1454500	23956.5		131000000	8.1		53072000	401
